# Sonication assisted microbiological diagnosis of implant-related infection caused by *Prevotella disiens* and *Staphylococcus epidermidis* in a patient with cranioplasty

**DOI:** 10.1186/s13104-015-1274-x

**Published:** 2015-07-17

**Authors:** Pavlos C Thomaidis, Angeliki Pantazatou, Spyros Kamariotis, Konstantinos Vlachos, George Roussos, Petros Panagiotou, Antonios Stylianakis

**Affiliations:** Laboratory of Implant Infections, Department of Microbiology, “KAT” General Hospital, 14561 Athens, Greece; Department of Microbiology, “Laikon” General Hospital, 11527 Athens, Greece; Neurosurgery Department, “KAT” General Hospital, 14561 Athens, Greece; Plastic Surgery Department, “KAT” General Hospital, 14561 Athens, Greece

**Keywords:** Sonication, Implants, Biofilm, Implant related infections, Anaerobes

## Abstract

**Background:**

Infections present a major complication of cranioplasty procedures and in many cases removal of the implant material becomes a necessity. Sonication of the artificial implant material has been used during the last years, in order to facilitate better diagnosis of these infections, nevertheless its use in cranial implant infections is still limited.

**Case presentation:**

A case of a 63-year-old Caucasian male patient who underwent a decompressive craniectomy, due to intracranial hemorrhage, and a consequent cranioplasty using an autogenic bone flap fixed by titanium clamps, is reported. After three unsuccessful cranioplasty efforts to repair a persistent skin defect, removing the bone flap and the titanium clamps was a necessity. Tissue and bone cultures were unable to reveal any microorganism whilst sonication of the removed titanium clamps and consequent culture of the resulting sonication liquid yielded *Prevotella disiens* and *Staphylococcus epidermidis*. The patient was treated with daptomycin and metronidazole until discharge and the skin defect was successfully repaired.

**Conclusion:**

The present case report indicates that the use of the sonication procedure assisted the microbiological diagnosis. This is the first known neurosurgical case of the implementation of the sonication procedure.

## Background

Post cranioplasty infection is a major complication (with a reported incidence of up to 10%), leading to increased morbidity and removal of the implant material becomes a necessity in many cases [1]. However, this figure may represent an underestimation as many cases may be misdiagnosed by conventional microbiological methods. The help of sonication in the microbiological diagnosis of cranial implant infections is still limited but can be a useful technique to improve diagnosis and treatment of these infections.

## Case presentation

A 63-year-old Caucasian male patient had a left parietal decompressive craniectomy due to intracranial hemorrhage, followed by cranioplasty. During cranioplasty at first place, an autogenic bone flap was fixed using titanium clamps (CranioFix, Aesculap, Germany) to repair the cranial defect.

Ten months later, due to a severe skin defect, he underwent a secondary cranioplasty effort, however with unsuccessful results. The patient reported a delayed wound healing with no symptoms of infection.

**Figure 1:**
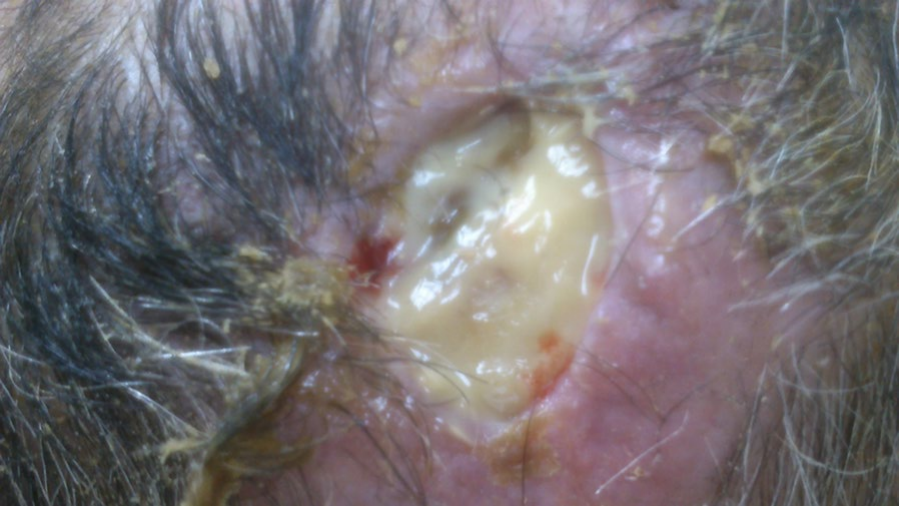
Patient during admission at KAT hospital.

**Figure 2:**
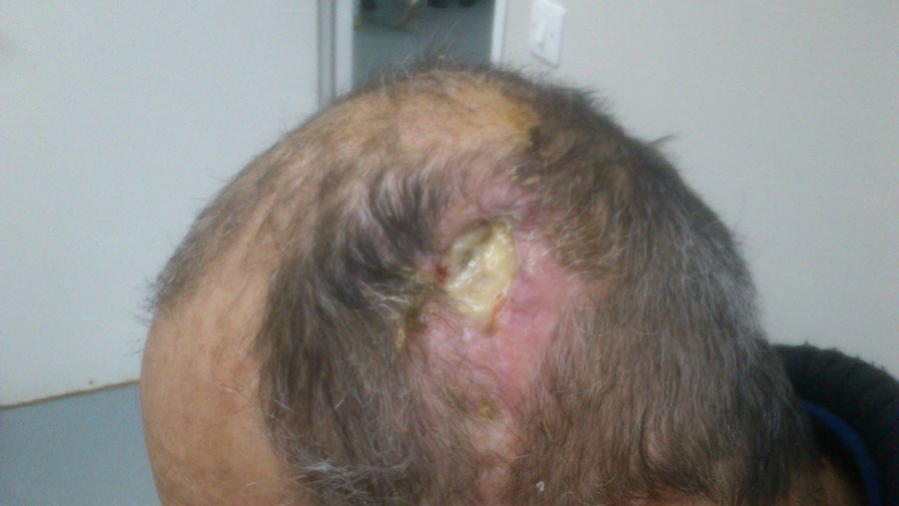
Patient during admission at KAT hospital.

Five months after the second cranioplasty he was admitted in KAT hospital for the programmed repair of the persistent skin defect. During clinical examination bone exposure was reported with no signs of infection and all cultures taken from the spot were negative (Figures 1, 2). Laboratory examination revealed normal levels of white blood cell count (WBC), C-reactive protein (CRP) and erythrocyte sedimentation rate (ESR). Teicoplanin (400 mg, IV, 1 × 2) was used as prophylaxis and a thorough surgical debridement was performed, without removing any cranioplasty materials. A skin flap was used to cover the cranial defect. On the day after the operation, he presented with fever (39°C), high ESR (84 mm/h), increased CRP (5 mg/dl) and normal WBC (6,950 × 10^9^ leucocytes/Lt). A parietal abscess was clinically identified, pus samples were collected for microbiological diagnosis and the patient was started empirically on daptomycin (20 mg/kg, per day, IV), ciprofloxacin (400 mg, IV, 1 × 2) and metronidazole (500 mg, IV, 1 × 3). Daptomycin dosage was higher than recommended due to the proximity of the infection to the central nervous system. Cultures were performed on 5% sheep blood agar, MacConkey agar, Sabouraud agar (media produced in-house) and anaerobe 5% sheep blood agar plates (Bioprepare, 19001, Keratea, Greece) and yielded methicillin-susceptible *Staphylococcus epidermidis.* The patient remained febrile until the fourth postoperative day, while the same antimicrobial treatment was continued for 2 weeks, until discharge. On the ninth postoperative day CRP was 1.39 mg/dl, ESR was 78 mm/h while WBC was normal (6,960 × 10^9^ leucocytes/Lt). The patient was afebrile upon discharge.

The patient was seen 3 weeks after discharge and samples were obtained from the operation site for microbiological diagnosis, revealing methicillin-resistant *Staphylococcus epidermidis* (daptomycin susceptible). Hence removal of the bone flap and the titanium clamps was decided. During surgery, tissue and bone samples, as well as the removed titanium clamps, were submitted for microbiological diagnosis. No repair of the bone defect was performed, considering the limited extend of craniectomy. The patient was started on daptomycin (700 mg, per day, IV) and metronidazole (500 mg, IV, 1 × 3).

The tissue and bone samples were homogenized and inoculated in 5% sheep blood agar, MacConkey agar, Sabouraud agar (media produced in-house) and anaerobe 5% sheep blood agar plates (Bioprepare). Cultures were incubated in ambient air, 5% CO_2_ and anaerobic conditions (according to the culture media types and the diagnostic protocols) for a total of 5 days. The titanium samples were sonicated, as described previously [2] using the ultrasound bath BactoSonic (Bandelin GmbH, Berlin, Germany), at a frequency of 40 kHz and power density of 0.22 W/cm^2^, as determined by a calibrated hydrophone (type 8103; Bruel and Kjær, Naerum, Denmark). A total of 100 μl of the sonication fluid were inoculated in the same culture media as the homogenized tissue and bone samples, and incubated under the same conditions, whilst slides were prepared for Gram-stain. Gram stain of the sonication fluid was negative. After incubation all tissue and bone samples were proven sterile. However, sonication fluid cultures yielded methicillin-resistant *Staphylococcus epidermidis* (daptomycin susceptible). Additionally, small, round and opaque colonies were detected on the third day of incubation on the anaerobe blood agar plate. Anaerobic growth was confirmed by aerotolerance testing. Gram staining of the isolate revealed short, slightly staining Gram-negative rods. Preliminary identification, using the special potency disk method [3] was suggestive of *Prevotella* genus (resistant to 1,000 μg kanamycin and 5 μg vancomycin disks and susceptible to 10 μg colistin disk). The strain was identified as *Prevotella disiens* using the BBL Crystal Anaerobe Identification System (Becton–Dickinson and Co, Sparks, MD 21152, USA). Susceptibility testing was performed using the gradient strip method and the E-test strips (bioMerieux, Marcy L’ Etoile, France) on brucella agar plates supplemented with 5% sheep blood, 1 mg/l vitamin K and 5 mg/l haemin, under anaerobic conditions, in a Bactron 1.5 Anaerobic Chamber (Cheldon Manufacturing, Cornelius, OR, USA), for 48 h. Susceptibility results were interpreted using CLSI [4] and EUCAST breakpoints [5]. The isolate was susceptible to all antimicrobials tested (MICs of piperacillin-tazobactam, cefoxitin, clindamycin, imipenem and metronidazole were <0.016, 0.064, 0.25, 0.016, 0.032 and 0.032 mg/l respectively). *Bacteroides fragilis* ATCC 25285 and *Bacteroides thetaiotaomicron* ATCC 29741 strains were used for quality control, whilst anaerobiosis was ensured with methylene blue strips and resazurine.

Antimicrobial chemotherapy with daptomycin and metronidazole continued until discharge, 25 days later. Postoperative recovery was uneventful and the patient remained afebrile and asymptomatic. The skin defect was successfully treated, while repair of the cranial defect is yet to be decided.

## Discussion

*Prevotella* spp. are obligately anaerobic, saccharolytic, non-motile, non-sporeforming, pleomorphic Gram-negative rods, showing various degrees of pigmentation [3]. They are found as members of the normal flora in the oral cavity, the gastrointestinal and upper respiratory tracts and urogenital area [3]. They have been mainly isolated from infections of these sites, however they have been present and considered as cause of infections in a variety of anatomic sites [3].

*Prevotella disiens* is a non-pigmented, saccharolytic and proteolytic strain, occasionally isolated from polymicrobial infections of the upper respiratory tract [6], central nervous system [7], urogenital [8] and oral [9, 10] tract. To the best of our knowledge the present case is the first documented implant-related infection due to a *Prevotella* spp.

The role of the biofilm is crucial for the development of device-related infections [11], which are difficult to be treated as bacteria are protected within the biofilm from antimicrobial agents and the host’s immune system [12]. Data indicate that the biofilm matrix is often multibacterial and different microorganisms can be attached on the implant surface [13] resulting in quorum sensing [14]. In the present case, despite the use of multiple broad spectrum antimicrobials, recovery was succeeded only after the removal of the cranioplasty materials, indicating a constant low-grade implant-related infection.

The sonication technique can disrupt the biofilm and release the pathogens into the sonication fluid, which is consequently cultured using conventional microbiological methods. Considering that the causative pathogen of a prosthetic-related infection can be situated exclusively on the surface of the prosthetic device, clustered within the biofilm, diagnosing the infection using multiple periprosthetic tissue cultures lacks of sensitivity. Sonication is also more capable to diagnose infection when the patient is under empirical antimicrobial therapy. Sensitivity and specificity of this method seems to be improved compared to the conventional periprosthetic tissue cultures. Sonication liquid culture is a faster method and more feasible in detecting polymicrobial infections compared to periprosthetic tissue culture. In our case conventional tissue and bone cultures were unable to reveal all the causative microorganisms of the implant-related infection. Sonication permits the isolation not only of the most frequent pathogens of the implant-related infections, such as *Staphylococcus* spp, but also of the highly fastidious ones [2]. Sonication fluid can be additionally processed using molecular techniques as well, improving sensitivity and specificity furthermore. This method is cost-effective, fast and easy to perform as does not requires special training.

## Conclusion

In the present case is highlighted the impact of sonication in the diagnosis of a multibacterial implant-related infection in a patient with cranioplasty. Repeated efforts to repair the persistent skin defect were proven unsuccessful, indicating that the removal of the implant materials was mandatory. Definitive microbiological diagnosis of infection was given only after the implementation of the sonication procedure, while conventional microbiological methods were ineffective. In that respect, our present experience agrees with those reports indicating that sonication can increase the sensitivity of the culture of artificial implanted materials and should be considered as a valid alternative technique.

## Abbreviations

WBC: white blood cell
CRP: C-reactive protein
ESR: erythrocyte sedimentation rate
MIC: minimum inhibitory concentration
CLSI: clinical and laboratory standards institute
ATCC: American type culture collection
